# The Modern Operation for Cataract

**Published:** 1871-08

**Authors:** 


The Modern Operation for Cataract: A Lecture delivered at the Harvard Medical School, April 5, 1871, with an analysis of sixty-one operations, by Hasket Derby, M.D. Boston: 1871—a pamplet of 23 pages.

